# Transmission potential of *Culex* and *Aedes* species for Madariaga virus, a member of the eastern equine encephalitis virus complex

**DOI:** 10.1371/journal.pntd.0013516

**Published:** 2026-05-12

**Authors:** Danilo de Carvalho-Leandro, Francisco C. Ferreira, Nadia Fernández Santos, William J. Sames, Yuexun Tian, Martial L. Ndeffo-Mbah, Erica A. Costa, Michael J. Turell, Gabriel L. Hamer, Tereza Magalhaes

**Affiliations:** 1 Department of Entomology, Texas A&M University, College Station, Texas, United States of America; 2 Colégio de Aplicação, Universidade Federal de Pernambuco, Recife, Brazil; 3 Instituto Politécnico Nacional, Centro de Biotecnología Genómica, Reynosa, Mexico; 4 Independent Researcher, Leakey, Texas, United States of America; 5 Department of Integrative Biosciences, College of Veterinary Medicine and Biomedical Sciences, Texas A&M University, College Station, Texas, United States of America; 6 Research Laboratory in Animal Virology, Veterinary School, Universidade Federal de Minas Gerais, Belo Horizonte, Brazil; 7 VectorID LLC, Frederick, Maryland, United States of America; The University of Hong Kong, CHINA

## Abstract

Madariaga virus (MADV), widely distributed in Latin America, can cause severe disease in humans and equids, yet key aspects of its transmission cycle remain unclear. To identify mosquitoes that could act as vectors of MADV, we assessed the vector competence of *Aedes aegypti*, *Ae. albopictus*, *Ae. taeniorhynchus*, *Culex tarsalis*, *Cx. coronator*, and *Cx. quinquefasciatus*, following oral exposure to MADV isolated in Panama (all species) or Brazil (*Ae. taeniorhynchus* only). We also evaluated temporal infection dynamics of MADV from Panama in *Ae. aegypti* and *Ae. albopictus*. MADV RNA and infectious virus were quantified in mosquito bodies, legs, and saliva. At 14 days post-exposure, five of the six species tested had virus detected in all biological sample types, indicating their potential to become infected with and transmit MADV. Conversely, *Cx. quinquefasciatus* was susceptible to midgut infection and dissemination but had no positive saliva samples, suggesting limited transmission potential. *Aedes taeniorhynchus* showed higher infection probabilities with MADV from Brazil compared with MADV from Panama. Time-course analysis revealed distinct infection dynamics in *Ae. aegypti* and *Ae. albopictus*, with infection increasing over time in *Ae. aegypti*, but peaking at 7 days post-exposure and then gradually declining in *Ae. albopictus*. Our findings indicate that MADV may be compatible with multiple mosquito species with broad geographic distributions, reinforce the need to investigate species- and strain-specific mosquito-virus interactions and their influence on arbovirus transmission dynamics, and support a potential role for *Ae. taeniorhynchus* as an amplification and bridge vector in endemic regions.

## Author summary

Madariaga virus (MADV) is a mosquito-borne virus found throughout Latin America that can cause severe disease in humans and equids, yet its mosquito vectors are not well defined. Identifying mosquitoes capable of transmitting MADV is important for understanding its transmission cycles and anticipating outbreaks. Here, we evaluated whether six mosquito species commonly found in the Americas – *Aedes aegypti*, *Ae. albopictus*, *Ae. taeniorhynchus*, *Culex coronator*, *Cx. quinquefasciatus*, and *Cx. tarsalis* – can become infected with MADV and potentially transmit it. Our results indicate that five of the species tested might be able to act as vectors, as demonstrated by the presence of infectious virus in their saliva, whereas *Cx. quinquefasciatus* showed no detectable virus in saliva despite becoming infected. We also evaluated temporal infection patterns in *Ae. aegypti* and *Ae. albopictus* and observed that infection increased over time in *Ae. aegypti*, but peaked and then declined in *Ae. albopictus*. Lastly, *Ae. taeniorhynchus* was more efficiently infected with a MADV strain from Brazil than with a strain from Panama. Overall, these findings suggest that MADV might be compatible with multiple mosquito species in the field and highlight the importance of mosquito-virus interactions at both species and strain levels.

## 1. Introduction

Madariaga virus (MADV), an encephalitic alphavirus belonging to the eastern equine encephalitis virus (EEEV) complex, is prevalent in Latin America and a potential emerging pathogen [1]. The virus circulates in enzootic cycles involving sylvatic mosquito vectors and vertebrate animals and can cause disease in equids and humans during spillover events. Case fatality rates among equines can reach >90% during epizootics [1], and severe and fatal human cases due to MADV infection have been documented [2–6].

The vectors of MADV remain unknown, and different mosquito species likely act as vectors across the various biomes where the virus circulates in Latin America [1]. Nonetheless, field and laboratory data have helped identify potential candidates. In the field, MADV has been isolated from several mosquito species, with isolations from *Culex* (*Melanoconion*) spp. being more frequent [1, 7–12]. The virus has also been detected in *Aedes taeniorhynchus* on more than one occasion, including during an equine epizootic in Brazil in 1960 [11,13], suggesting this species may act as an amplification and bridge vector during outbreaks in certain regions. A limitation in these field studies is the lack of information about the physiological status of mosquitoes (i.e., whether females were engorged or not), raising the possibility that virus detection could have been from traces of infected host’s blood and not from infected mosquito tissues.

As for laboratory studies, to date only one vector competence study has been published on MADV. In that study, mosquito species collected in the Peruvian Amazon were tested for their ability to transmit the virus using a live animal model [14]. The following species were shown to be competent vectors: *Cx.* (*Mel.*) *pedroi*, *Ae.* (*Ochlerotatus*) *fulvus*, *Psorophora* (*Janthinosoma*) *albigenu*, *Ps.* (*Grabhamia*) *cingulata*, *Ae.* (*Och.*) *serratus*, and *Ps.* (*Jan.*) *ferox*.

Given the limited data on the identification of MADV vectors, we assessed the ability of selected mosquito species – most of which had not been previously tested – to transmit the virus following oral exposure. Species selection included one or more of the following criteria: 1) high abundance across various geographic regions within the Americas; 2) known role as a vector of arboviruses; 3) feeding habit on mammalian hosts indicating ability to act as bridge vectors; 4) evidence for having a potential role in enzootic or epizootic MADV transmission; and 5) experimental feasibility. The species tested were *Cx. coronator*, *Cx. quinquefasciatus*, *Cx. tarsalis*, *Ae. aegypti*, *Ae. albopictus*, and *Ae. taeniorhynchus*.

## 2. Methods

### 2.1. Mosquitoes

The vector competence for MADV was evaluated in six mosquito species: *Aedes aegypti*, *Ae. albopictus*, *Ae. taeniorhynchus*, *Culex coronator*, *Cx. tarsalis*, and *Cx. quinquefasciatus*.

*Aedes aegypti* and *Ae. albopictus* eggs were collected in September 2023 in Hidalgo County and Brazos County, Texas, respectively, using ovitraps. Adult *Ae. taeniorhynchus* were collected in August 2024 in Hidalgo County, Texas, using modified CDC light traps. *Culex coronator* larvae were collected in October 2024 in Leakey, Texas, using a larval dipper. *Culex tarsalis* and *Cx. quinquefasciatus* were obtained from established colonies maintained at Colorado State University. All specimens were transported to insectaries at Texas A&M University and reared under controlled conditions (28^o^C, 70% humidity, 12:12 h light/dark cycle) with *ad libitum* access to 10% sucrose and water. Adults of *Ae. taeniorhynchus* and *Cx. coronator* parental generation (F0), and F2-F3 adults of *Ae. aegypti* and *Ae. albopictus* were used in the experiments. *Culex tarsalis* and *Cx. quinquefasciatus* adult females used in the experiments were of unknown generation. All mosquitoes were 4–6 days old at the time of virus exposure, except for *Ae. taeniorhynchus*, which were collected as adults from the field and used directly in the experiments, therefore these were of unknown age.

Mosquito specimens brought from the field (F0) and the first filial generation (F1) were reared in the Arthropod Containment Level (ACL)-2 room; after that, mosquitoes were reared in ACL-1 rooms.

### 2.2. Viruses

MADV-PAN (Lineage III; GenBank: KJ469648), isolated from a horse during a 2010 outbreak in Panama, was provided by the World Reference Center for Emerging Viruses and Arboviruses at the University of Texas Medical Branch, and used to expose all mosquito species. Passage 3 MADV-PAN was received lyophilized and subsequently resuspended in cell culture medium. MADV-BR (Lineage III; GenBank: MZ389692), isolated by our group in 2019 from the central nervous system of a deceased horse during an epizootic in Northeast Brazil [15], was used to expose *Ae. taeniorhynchus* only. Passage 9 MADV-BR was received as frozen aliquots. The two strains share 95% nucleotide and 99% amino acid identity.

MADV-PAN and MADV-BR were propagated in Vero cells (CCL-81, ATCC) maintained at 37^o^C with 5% CO_2_ in complete Dulbecco’s Modified Eagle Medium (DMEM) containing 5% fetal bovine serum (FBS). Virus stocks were generated by infecting 80% confluent monolayers and harvesting cells and supernatant at 36 h post-infection, when cytopathic effect reached 80–90%. The collected material was centrifuged (1,000xg, 5 min, 4^o^C), and the supernatant was aliquoted and stored at -80^o^C. Final stock titers were 1.6 x 10^7^ plaque-forming units (PFU)/mL for MADV-PAN (passage 5) and 4.0 x 10^7^ PFU/mL for MADV-BR (passage 11). For mosquito infections, viruses were propagated using the same methodology, but fresh supernatant was mixed with blood immediately after centrifugation.

Because MADV is a Biosafety Level 3 (BSL-3) pathogen, all procedures involving the virus were conducted in BSL-3 and ACL-3 laboratories under protocol IBC2022–075, approved by the Texas A&M University Institutional Biosafety Office.

### 2.3. Mosquito exposure

Adult female mosquitoes were transferred to the ACL-3 and acclimated for 1–2 days in a growth chamber (28^o^C, 70% humidity, 12:12 h light/dark cycle). Groups of 200–400 mosquitoes were starved for 24 h, then offered a 1:1 mixture of defibrinated calf blood (Colorado Serum Company) and freshly harvested MADV-PAN or MADV-BR through a glass membrane feeder apparatus. Mosquitoes were allowed to feed for 30 min, after which engorged females were sorted, transferred to 473 mm^3^-cartons, and maintained with 10% sucrose and water for up to 21 days. An aliquot of the blood:virus mixture was kept at 37^o^C during feeding and later stored at -80^o^C for bloodmeal titration. Two independent experimental replicates were performed per mosquito species, and per *Ae. taeniorhynchus* exposure with each virus strain.

### 2.4. Sample collection

Thirteen to 30 MADV-exposed mosquitoes per group, and per time point for *Ae. aegypti* and *Ae. albopictus*, were sampled in each experimental replicate. Bodies, legs (all six legs from each mosquito were combined into a single sample), and saliva were collected and stored individually for RNA and infectious virus quantification. Collections were performed at 14 days post-exposure (dpe) for all species, and additionally at 3, 7, and 21 days for *Ae. aegypti* and *Ae. albopictus*. For sample collection, females were anesthetized (2^o^C), and legs were removed and placed in microcentrifuge tubes with 250 µL mosquito diluent (PBS with 20% FBS, antibiotics, and antimycotic) and borosilicate beads. Wings were discarded. Saliva was collected at room temperature by forced salivation, by inserting the proboscis into glass capillaries containing immersion oil type B. After 30 min of salivation, capillaries were individually transferred to microcentrifuge tubes with 80 µL mosquito diluent and centrifuged (11,000xg, 3 min, 2^o^C). Mosquito bodies were then placed in microcentrifuge tubes with 250 µL mosquito diluent and borosilicate beads. Leg and body samples were homogenized for 1 min at 30 Hz in a TissueLyzer II. All samples were stored at -80^o^C. For molecular and plaque assays, samples were thawed and centrifuged (11,000xg, 3 min, 2^o^C).

### 2.5. RNA extraction and reverse transcription quantitative PCR (RT-qPCR)

RNA was extracted from virus stocks, bloodmeal samples, and mosquito biological samples using the Mag-Bind Viral DNA/RNA Kit (Omega Bio-tek) on a KingFisher Flex System with 96-deep well head, following the manufacturers’ instructions. A 50-µL sample volume was used, and RNA was eluted in 50 µL of ultra-pure water. For RNA extraction, virus stocks were first serially diluted in 10-fold dilutions (undiluted to 10^-9^) in 5% DMEM, and bloodmeal and mosquito samples were processed after stored material was thawed and centrifuged.

The following primers and probe targeting MADV nsP2 (nt 1776–1892) were designed using IDT PrimerQuest: forward (5’-3’) – GGCTGAACAGGTGCTAGTTAT; reverse (5’-3’) – CTATTCCAATCCCGGACTTTCA; probe (5’-3’) – CGCGCCGGTAGGTACAAAGTAGAA (6-FAM/ZEN/3’ IB FQ). Amplicon size was 117 bp. Reactions were prepared using the iTaq Universal Probes One-Step Kit (Bio-Rad). Thermocycling was performed on a Bio-Rad CFX96 under the following conditions: 50^o^C for 10 min, 95^o^C for 2 min; 40 cycles of 95^o^C for 15 sec and 60^o^C for 30 sec. Each run included a MADV RNA standard curve (10^-2^-10^-6^) and a no-template (water) control. A cycle threshold (Ct) ≤38 was considered positive.

### 2.6. Plaque assays

To confirm the presence of infectious virus in the samples, all saliva and 50% of body and leg RT-qPCR-positive samples were tested by plaque assay. Virus stocks and bloodmeal contents were also assayed. For that, Vero cells at 80% confluency in 24-well plates were inoculated with samples (150 µL-volume, with samples brought up to 150 µL with plain DMEM when the original sample volume was lower), followed by 1 h adsorption and subsequent addition of overlay medium (1:1 Tragacanth medium:2x 8% DMEM). After a 3-day incubation at 5% CO_2_ and 37^o^C, the overlay medium was removed, and the monolayer was fixed and stained with 0.1% crystal violet in 20% ethanol. Plates were allowed to dry and plaques were counted to calculate PFU/mL based on dilution factors. Bloodmeal samples were plated in duplicate for each dilution (10^-2^-10^-6^); body and leg samples were plated in one replicate per dilution (10^-2^ and 10^-3^); and saliva samples (post-RNA extraction, approximately 15 µL, brought to a final volume of 150 µL with DMEM) were plated at a 10^-1^ dilution in a single well. Negative controls (medium only) were included in each plate. Fifteen RT-qPCR-negative samples per tissue type were randomly selected and also assayed.

### 2.7. Data analysis

All data analyses were conducted in SAS Studio, whereas graphs were generated in GraphPad Prism 10.5.0.

#### 2.7.1. Midgut infection, dissemination, and transmission rates.

First, midgut infection, dissemination, and transmission rates were calculated. Midgut infection rates were calculated as the number of RT-qPCR-positive body samples divided by the total number of body samples tested (per species, timepoint, and replicate), multiplied by 100. Body samples were used as a proxy for midgut infection and the term “midgut infection” is used hereafter to refer to body data in this analysis. Dissemination rates were calculated in the same manner but using data from leg samples. Legs were used as a proxy for dissemination because detection of virus in the legs indicates that the virus has escaped the midgut and entered the hemolymph, enabling spread to secondary tissues. Transmission rates were calculated in the same manner but using data from saliva samples. The use of body, legs, and saliva samples as proxies for midgut infection, dissemination, and transmission is well established in vector competence studies [16,17]. No statistical tests were applied here, as differences were assessed using regression models.

#### 2.7.2. Infection probabilities of body, legs and saliva samples.

For a more robust statistical analysis and to account for bloodmeal titers and experimental replicates, infection status probabilities were estimated for each sample type (body, legs, and saliva) using logistic regression models. In these analyses, ‘infection probability’ was calculated for each type of biological sample using the same RT-qPCR data used to calculate midgut infection, dissemination, and transmission rates. Models included fixed effects according to the specific analysis (‘mosquito species’, ‘virus strain’, ‘dpe’, and ‘mosquito species x dpe’ interaction) and covariates (‘bloodmeal titer’ and ‘replicate’), and were fitted using the GLIMMIX procedure. Bloodmeal titers were log_10_-transformed for the analyses. Least-square means and 95% confidence intervals (CIs) were estimated to derive infection probabilities for each sample type and mosquito species and, for *Ae. aegypti* and *Ae. albopictus*, at each timepoint. Pairwise comparisons between groups were conducted using odds ratios (ORs) derived from the same models, with significance set at p < 0.5. ORs < 1 indicate lower odds of infection, whereas ORs > 1 indicate higher odds, relative to the reference group.

Models were applied to: (i) compare MADV-PAN infection probabilities in body, legs and saliva across species at 14 dpe (‘mosquito species’ as fixed effect; reference species: *Ae. aegypti*); (ii) compare MADV-PAN and MADV-BR infection probabilities in body, legs and saliva of *Ae. taeniorhynchus* at 14 dpe (‘virus strain’ as fixed effect; reference strain: MADV-PAN); and (iii) assess the temporal variation in MADV-PAN infection probabilities in body, legs, and saliva across timepoints in *Ae. aegypti* and *Ae. albopictus* (‘mosquito species’, ‘dpe’, and ‘mosquito species x dpe’ as fixed effects; references: *Ae. aegypti*, 3 dpe). In (i), groups with no positive saliva samples were excluded from the models due to lack of variation in the outcome, which precludes reliable estimation in logistic regression. In these cases, differences between species with zero positive samples and other species were assessed using Fisher’s exact tests with FDR and Bonferroni correction. In (iii), time points with zero positives were retained to preserve model structure, as inclusion of the time variable and its interaction with mosquito species required maintaining all levels of the factor for appropriate comparisons across time points.

In (iii), models accounting for repeated measures were not used because the data were not longitudinal, as different mosquitoes were used for sample collection at each timepoint.

#### 2.7.3. MADV titers in body, legs and saliva samples.

Virus titers (PFU/mL) were log_10_-transformed and least-square means and 95% confidence intervals (CIs) were obtained using generalized linear models.

## 3. Results

### 3.1. Bloodmeal titers

Bloodmeal titers for MADV-PAN ranged from 1.1 x 10^5^ to 5.8 x 10^7^ PFU/mL across all experiments. Bloodmeal titers for MADV-BR ranged from 2.4 x 10^7^ to 4 x 10^7^ PFU/mL (Table 1).

**Table 1:** Numbers of mosquito body, leg, and saliva samples that tested positive through reverse transcription quantitative PCR using Madariaga virus-specific primers and probe.

Mosquito species	Replicate	MADV lineage	Bloodmeal titer (PFU/mL)*	Dpe	Midgut infection rate	Dissemination rate	Transmission rate
					Positive body samples/ Total body samples tested (%)	Positive leg samples/ Total leg samples tested (%)	Positive saliva samples/ Total saliva samples tested (%)
*Aedes aegypti*	1	MADV-PAN	2.0 x 10^7^	3	6/20 (30%)	0/20 (0%)	0/20 (0%)
				7	6/20 (30%)	2/20 (10%)	1/20 (5%)
				14	13/20 (65%)	7/20 (35%)	5/20 (25%)
				21	18/20 (90%)	11/20 (55%)	8/20 (40%)
	2	MADV-PAN	1.3 x 10^5^	3	7/20 (35%)	2/20 (10%)	0/20 (0%)
				7	7/20 (35%)	3/20 (15%)	1/20 (5%)
				14	12/20 (60%)	7/20 (35%)	3/20 (15%)
				21	15/20 (75%)	9/20 (45%)	5/20 (25%)
			Total**:		**25/40 (63%)**	**14/40 (35%)**	**8/40 (20%)**
*Aedes albopictus*	1	MADV-PAN	2.0 x 10^7^	3	4/20 (20%)	4/20 (20%)	0/20 (0%)
				7	16/20 (80%)	9/20 (45%)	4/20 (20%)
				14	6/20 (30%)	4/20 (20%)	2/20 (10%)
				21	3/20 (15%)	2/20 (10%)	0/20 (0%)
	2	MADV-PAN	1.3 x 10^5^	3	7/20 (35%)	7/20 (35%)	2/20 (10%)
				7	10/20 (50%)	2/20 (10%)	2/20 (10%)
				14	6/20 (30%)	3/20 (15%)	2/20 (10%)
				21	1/20 (1%)	0/20 (0%)	0/20 (0%)
			Total**:		12/40 (30%)**	7/40 (18%)**	4/40 (10%)**
*Aedes taeniorhynchus*	1	MADV-PAN	5.8 x 10^7^	14	6/15 (40%)	2/15 (13%)	1/15 (7%)
	2		1.8 x 10^7^	14	5/13 (39%)	5/13 (38%)	2/13 (15%)
			**Total:**		**11/28 (39%)**	**7/28 (25%)**	**3/28 (11%)**
	1	MADV-BR	2.4 x 10^7^	14	21/30 (70%)	15/30 (50%)	8/30 (27%)
	2		4.0 x 10^7^	14	12/13 (92%)	6/13 (46%)	5/13 (38%)
			**Total:**		**33/43 (77%)**	**21/43 (49%)**	**13/43 (30%)**
*Culex coronator*	1	MADV-PAN	4.9 x 10^6^	14	4/20 (20%)	3/20 (15%)	2/20 (10%)
	2		1.11 x 10^5^	14	5/18 (28%)	4/18 (22%)	3/18 (17%)
			**Total:**		**9/38 (24%)**	**7/38 (18%)**	**5/38 (13%)**
*Culex tarsalis*	1	MADV-PAN	2.7 x 10^7^	14	9/30 (30%)	8/30 (27%)	4/30 (13%)
	2		4.4 x 10^6^	14	11/30 (37%)	10/30 (33%)	3/30 (10%)
			**Total**		**20.60 (33%)**	**18/60 (30%)**	**7/60 (12%)**
*Culex quinquefasciatus*	1	MADV-PAN	2.7 x 10^7^	14	4/30 (13%)	0/30 (0%)	0/30 (0%)
	2		4.4 x 10^6^	14	5/30 (17%)	2/30 (7%)	0/30 (0%)
			**Total:**		**9/60 (15%)**	**2/60 (3%)**	**0/60 (0%)**

Dpe: days-post exposure. MADV-PAN: Madariaga virus strain Panama. MADV-BR: Madariaga virus strains Brazil. PFU/mL: plaque forming units per mL. Midgut infection, Dissemination and Transmission rates are based on the number of body, leg, and saliva samples testing positive in the reverse-transcription quantitative PCR using primers and probe specific for Madariaga virus, divided by the total number of the respective samples tested, multiplied by 100.

*Bloodmeal titers were identical among species within a replicate when multiple species were fed concomitantly on the same infectious bloodmeal.

**Data for Day 14 only, for comparison with the other species.

### 3.2. MADV-PAN infection, dissemination, and transmission rates across mosquito species

All six species were susceptible to midgut infection and exhibited virus dissemination. Virus was detected in the saliva of all species, except *Cx. quinquefasciatus*. *Aedes aegypti* had the highest midgut infection rate (53%, 84/160) (Table 1).

In *Ae. taeniorhynchus*, rates were overall higher following exposure to MADV-BR than to MADV-PAN (Table 1).

Temporal patterns in *Ae. aegypti* and *Ae. albopictus* revealed distinct trends. In *Ae. aegypti*, midgut infection, dissemination and transmission rates increased over time, respectively reaching 82.5%, 50% and 32.5% at 21 days post-exposure (dpe). In contrast, *Ae. albopictus* showed decreasing rates after 7 dpe, with midgut infection declining to 10%, dissemination to 5%, and transmission to 0% by 21 dpe (Table 1).

### 3.3. MADV-PAN infection probabilities in body, legs, and saliva across mosquito species

In addition to calculating midgut infection, dissemination, and transmission rates, we modeled infection probabilities in body, leg, and saliva samples to evaluate differences across groups while accounting for bloodmeal titers and experimental variation.

In the final models for body, leg, and saliva samples, ‘bloodmeal titer’ was retained as a covariate, whereas ‘replicate’ was removed, as it did not predict the outcome. ‘Mosquito species’ significantly predicted infection status in both body (p < 0.001) and leg (p = 0.008) models.

**Fig 1:**
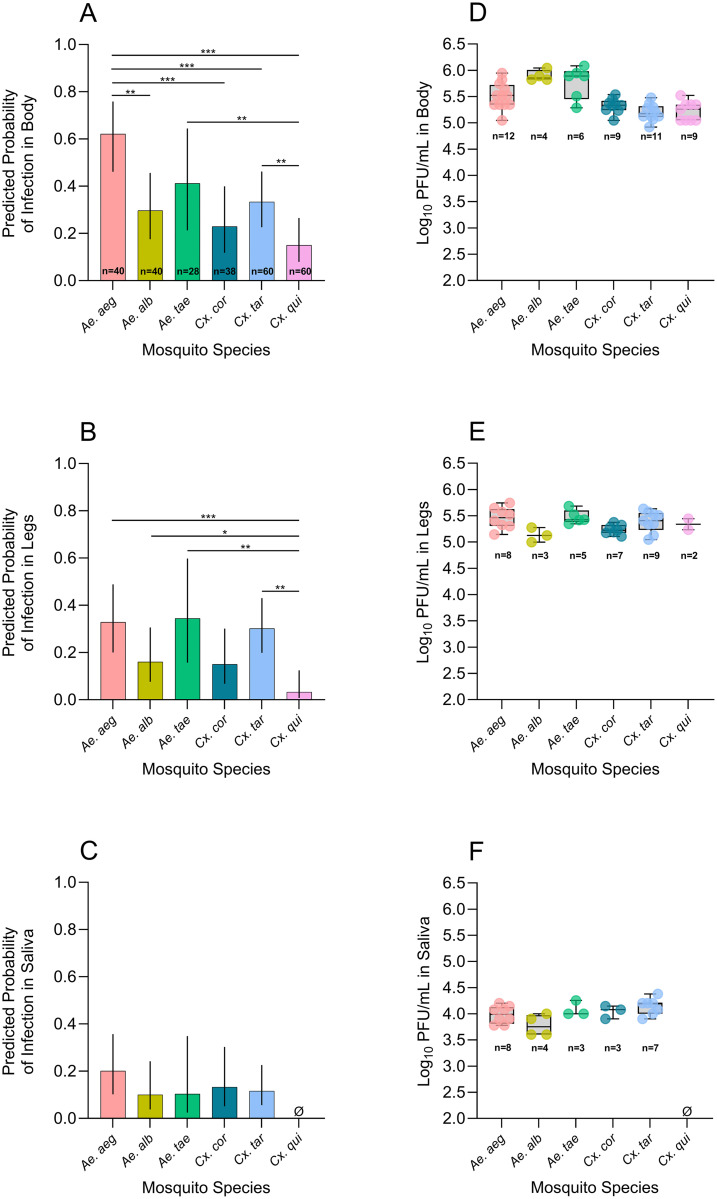
Predicted infection probabilities of Madariaga virus strain Panama (MADV-PAN) derived from regression models in body (A), legs (B), and saliva (C), and log_10_-transformed MADV-PAN titers in body (D), legs (E), and saliva (F) samples collected from distinct mosquito species at 14 days-post exposure. In A-C, the fixed effect was ‘mosquito species’, models were adjusted for ‘bloodmeal titer’, and two experimental replicates per mosquito species were accounted for; the total number of mosquitoes dissected per species across both replicates is shown in panel A, and vertical lines indicate 95% confidence intervals. In D-F, the number of samples tested is shown below each group, and box and whisker plots indicate the median and interquartile range – all of the saliva samples and 50% of body and legs testing positive in the molecular assay were tested in plaque assays. *Ae. aeg*: *Aedes aegypti*; *Ae. alb*: *Ae. albopictus*; *Ae. tae*: *Ae. taeniorhynchus*; *Cx. cor*: *Culex coronator*; *Cx. tar*: *Cx tarsalis*; *Cx. qui*: *Cx. quinquefasciatus*. In (C), data from Cx. *quinquefasciatus* was removed from the model as this species had zero positive saliva samples, leading to complete data separation. In (F), *Cx. quinquefasciatus* data is absent as there were no positive saliva samples. Bars with asterisks in A-C indicate the level of statistical significance between the two groups located at the ends of each bar, based on odds ratio pairwise comparisons: ^*^p < 0.05, ^**^p < 0.001, ^***^p < 0.0001. PFU/mL: plaque forming units per mL.

*Aedes aegypti* had the highest predicted body infection probability, followed by *Ae. taeniorhynchus*, *Cx. tarsalis*, *Ae. albopictus*, *Cx. coronator*, and *Cx. quinquefasciatus* (Fig 1A, S1 Table). ORs derived from pairwise comparisons indicated significant differences in body infection probability across several species. Compared to *Ae. aegypti*, body infection probability was significantly lower in *Ae. albopictus* (OR=0.26, p = 0.0046), *Cx. coronator* (OR=0.18, p = 0.001), *Cx. quinquefasciatus* (OR=0.11, p < 0.0001), and *Cx. tarsalis* (OR=0.31, p = 0.0063). In addition, *Cx. quinquefasciatus* also had significantly lower body infection probability than *Cx. tarsalis* (OR=0.35, p = 0.0225) (S2 Table).

Leg infection probabilities were highest in *Ae. aegypti*, *Ae. taeniorhynchus*, and *Cx. tarsalis*, and lowest in *Cx. quinquefasciatus* (Fig 1B, S1 Table). Pairwise comparisons showed significantly higher odds of leg infection in *Ae. albopictus* (OR=5.56, p = 0.0407) and *Ae. taeniorhynchus* (OR=15.31, p = 0.0026) compared to *Cx. quinquefasciatus*, and significantly lower odds were observed for *Cx. quinquefasciatus* relative to *Cx. tarsalis* (OR=0.08, p = 0.0012) and *Ae. aegypti* (OR=0.07, p = 0.001) (S2 Table).

In the saliva model, from which *Cx. quinquefasciatus* was excluded due to the absence of positive saliva samples, ‘mosquito species’ was not a significant predictor of infection status, although *Ae. aegypti* showed a trend toward higher saliva infection probabilities (Fig 1C, S1 Table). Pairwise comparisons from the model did not identify significant differences among species (S2 Table).

To enable comparison of *Cx. quinquefasciatus* saliva data with that of other species, Fisher’s exact tests were performed with Bonferroni or FDR correction. Under Bonferroni adjustment, the proportion of positive saliva samples in *Cx. quinquefasciatus* was significantly lower than in *Ae. aegypti* (p = 0.0020) and *Cx. coronator* (p = 0.0370). Under FDR correction, *Cx. quinquefasciatus* had significantly lower saliva positivity than all other species (adjusted p-values ranging from 0.0020 to 0.0299) (S3 Table).

### 3.4. MADV-PAN and MADV-BR infection probabilities in *Ae. taeniorhynchus*

**Fig 2:**
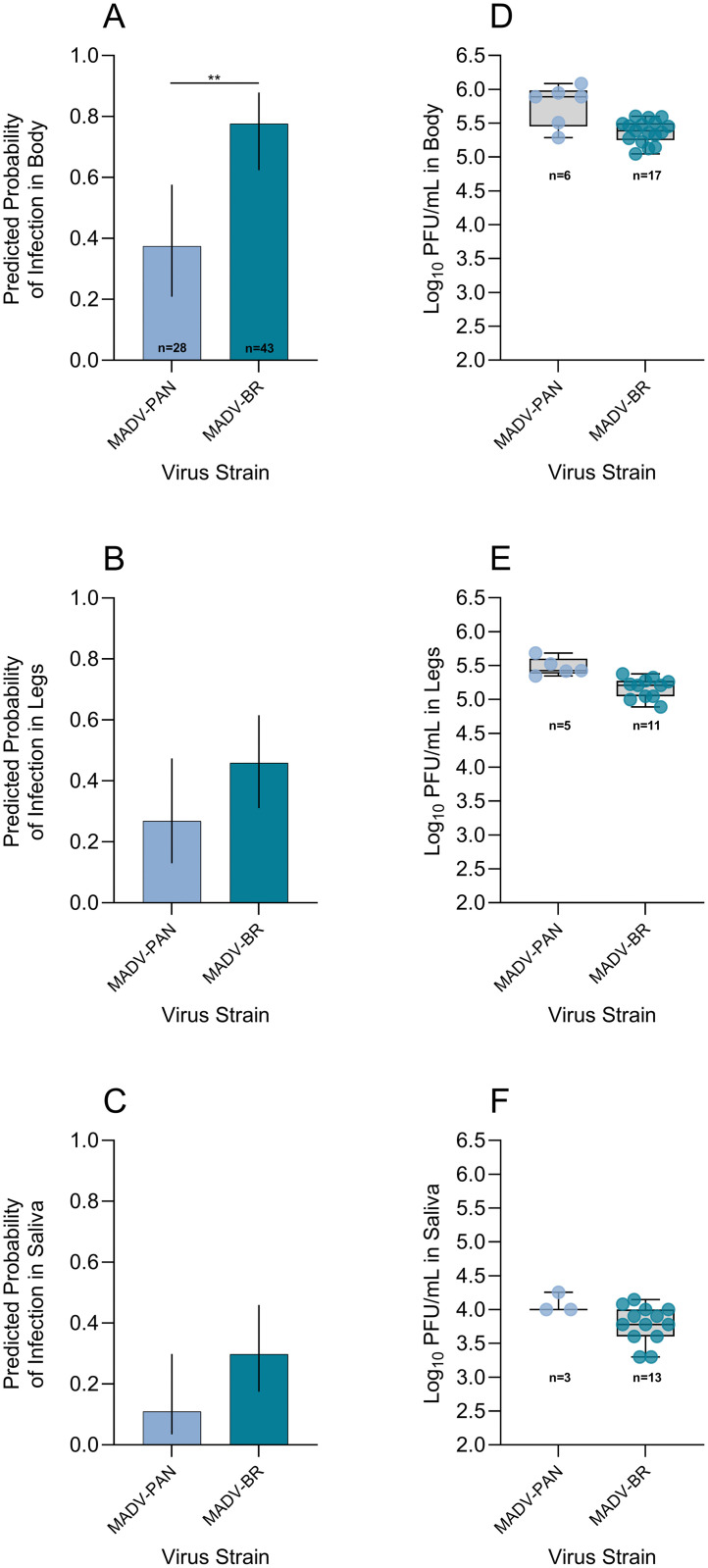
Predicted infection probabilities of Madariaga virus strain Panama (MADV-PAN) and Madariaga virus strain Brazil (MADV-BR) derived from regression models in body (A), legs (B), and saliva (C), and log_10_-transformed MADV-PAN and MADV-BR titers in body (D), legs (E), and saliva (F) samples collected from *Aedes taeniorhynchus* at 14 days-post exposure. In A-C, the fixed effect was ‘virus strain’, models were adjusted for ‘bloodmeal titer’, and two experimental replicates per virus strain were accounted for; the total number of mosquitoes dissected per species across both replicates is shown in panel A, and vertical lines indicate 95% confidence intervals. In D-F, the number of samples tested is shown below each group, and box and whisker plots indicate the median and interquartile range – all of the saliva samples and 50% of body and legs testing positive in the molecular assay were tested in the plaque assay. Bars with asterisks in A-C indicate the level of statistical significance between the two groups located at the ends of each bar, based on odds ratio pairwise comparisons: ^*^p < 0.05, ^**^p < 0.001, ^***^p < 0.0001. PFU/mL: plaque forming units per mL.

We compared infection probabilities in *Ae. taeniorhynchus* exposed to MADV-PAN and MADV-BR to assess differences in mosquito infection outcomes between the two virus strains. In the final models, ‘bloodmeal titer’ was retained as a covariate, whereas ‘replicate’ was excluded. ‘Virus strain’ was a significant predictor of infection status in body samples, with mosquitoes exposed to MADV-BR showing higher predicted body infection probabilities (OR=5.8, p = 0.0030) than those exposed to MADV-PAN. Although ‘virus strain’ was not a significant predictor in the leg and saliva models, there was a trend toward higher predicted infection probabilities in mosquitoes exposed to MADV-BR in these biological samples (leg: OR=2.32, p = 0.1326; saliva: OR=3.45, p = 0.0946) compared with MADV-PAN (Fig 2A - 2C, S4 and S5 Tables).

### 3.5. Temporal MADV-PAN infection probability in body, legs and saliva of *Ae.* *aegypti* and *Ae. albopictus*

To examine the temporal variation in infection outcomes in *Ae. aegypti* and *Ae. albopictus*, we modeled MADV-PAN infection probabilities in body, legs, and saliva samples across four timepoints. The final models for body, leg, and saliva data retained ‘bloodmeal titer’ as a covariate, whereas ‘replicate’ was excluded.

**Fig 3:**
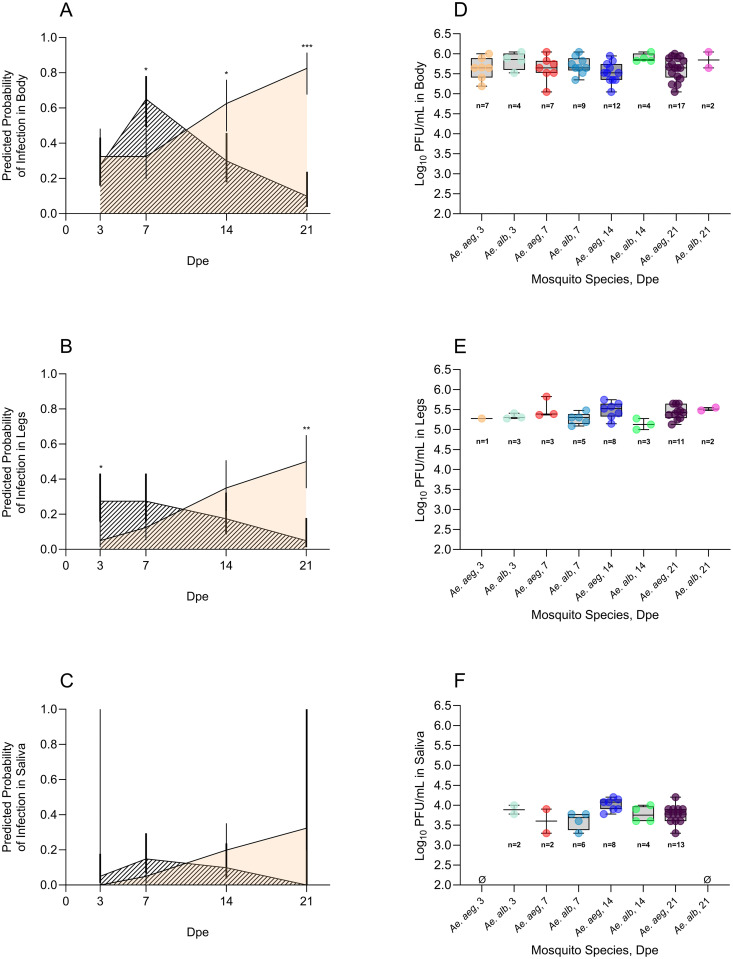
Predicted infection probabilities of Madariaga virus strain Panama (MADV-PAN) derived from regression models in body (A), legs (B), and saliva (C), and log_10_-transformed MADV-PAN titers in body(D), legs (E), and saliva (F) samples collected from *Aedes aegypti* (*Ae. aeg*; orange shading) and *Ae. albopictus* (*Ae. alb*; diagonal line shading) at 3, 7, 14, and 21 days-post exposure (dpe). In A-C, 20 individual mosquitoes were dissected per species per time point, the fixed effects were ‘mosquito species’, ‘dpe’, and ‘mosquito species x dpe’ interaction, models were adjusted for ‘bloodmeal titer’, and two experimental replicates per virus strain were accounted for. Vertical lines indicate 95% confidence intervals, and asterisks indicate the level of statistical significance between the two groups at a given time point, based on odds ratio pairwise comparisons: *p < 0.05, **p < 0.001, ***p < 0.0001. The wide confidence intervals in (C) reflect the absence of positive saliva samples in *Ae. aegypti* at 3 dpe and *Ae. albopictus* at 21 dpe – these groups were retained because the model included a ‘mosquito species x dpe’ interaction, and variation at other time points allowed estimation. In D-F, box and whisker plots indicate the median and interquartile range of PFU/mL; the number of samples tested is shown below each group – all of the saliva samples and 50% of body and legs testing positive in the molecular assay were tested in the plaque assay. PFU/mL: plaque forming units per mL.

In the body model, ‘mosquito species’ (p < 0.001) and ‘mosquito species x dpe’ interaction (p < 0.0001) were significant predictors of infection probability, while ‘dpe’ alone was not (p = 0.1). In *Ae. aegypti*, predicted body infection probabilities increased over time. In *Ae. albopictus*, infection probabilities increased from 3 to 7 dpe and declined thereafter (Fig 3A, S6 Table). Significant differences between species were observed at 7, 14, and 21 dpe, with *Ae. albopictus* showing higher odds of body infection at 7 dpe (OR=3.87, p = 0.0046), but lower odds at 14 dpe (OR=0.26, p = 0.0045) and 21 dpe (OR=0.02, p < 0.0001) compared to *Ae. aegypti* (S7 Table).

In the leg model, only ‘mosquito species x dpe’ interaction was a significant predictor of infection (p < 0.0001). Trends were similar to those in body samples, with increasing infection probabilities over time in *Ae. aegypti*, and a peak at 7 dpe followed by a decline at later timepoints in *Ae. albopictus* (Fig 3B, S6 Table). Significant differences between species were observed at 3 and 21 dpe, with *Ae. albopictus* showing higher odds of leg infection at 3 dpe (OR=7.23, p = 0.0149) and lower odds at 21 dpe (OR=0.05, p = 0.0002) compared to *Ae. aegypti* (S7 Table).

In the saliva model, none of the fixed effects (‘mosquito species’, ‘dpe’, or ‘mosquito species x dpe’) were significant predictors of infection status. Nevertheless, trends in predicted infection probabilities between *Ae. aegypti* and *Ae. albopictus* were similar to those observed in body and leg samples, with higher odds in *Ae. albopictus* at 7 dpe (OR=3.36, p = 0.155) and lower odds at 14 dpe (OR=0.44, p = 0.2183) compared to *Ae. aegypti*, although these differences were not statistically significant (Fig 3C, S6 and S7 Tables).

### 3.6. MADV titers

To assess the presence of infectious virus in the samples, plaque assays were performed on a subset of RT-qPCR-positive body and leg samples and on all RT-qPCR-positive saliva samples. In addition, a subset of RT-qPCR-negative samples was included as negative controls. Plaques were detected in all RT-qPCR-positive body, leg and saliva samples tested by plaque assay, with the exception of one *Ae. albopictus* saliva sample collected at 7 dpe (replicate 2). None of the 45 RT-qPCR-negative samples yielded plaques.

#### 3.6.1. MADV-PAN titers in body, legs, and saliva across mosquito species.

Mean MADV-PAN titers ranged from 5.2-5.9 log_10_ PFU/mL in body samples and 5.1-5.5 log_10_ PFU/mL in leg samples (Fig 1D-1E, S8 Table). Saliva PFU counts ranged from 1 to 12 per well at a 10^-1^ dilution. Given that an estimated 0.5-2 µL of saliva was obtained from mosquitoes and diluted in 80 µL of medium, and 50 µL of this mixture was used for RNA extraction, with additional volume loss during handling, the remaining sample volume (~15 µL) used for plaque assays represents <1 µL of actual saliva, resulting in estimated titers of 3.7-4.3 log_10_ PFU/mL across competent species (Fig 1F, S8 Table).

#### 3.6.2. MADV-PAN and MADV-BR titers in *Ae.* *taeniorhynchus* body, legs and saliva.

Mean MADV-PAN and MADV-BR titers in *Ae. taeniorhynchus* body samples were 5.8 and 5.3 log_10_ PFU/mL, respectively (Fig 2D, S9 Table). In leg samples, mean titers were 5.5 and 5.2 log_10_ PFU/mL for MADV-PAN and MADV-BR, respectively (Fig 2E, S9 Table). In saliva, mean titers for MADV-PAN and MADV-BR were 4.1 and 3.8 log_10_ PFU/mL, respectively (Fig 2F, S9 Table).

#### 3.6.3. MADV-PAN titers in *Ae.* *aegypti* and *Ae.* *albopictus* over time.

MADV-PAN titers in *Ae. aegypti* and *Ae. albopictus* showed limited variation across time points in body and leg samples. Titers in body samples ranged from 5.5 to 5.6 log_10_ PFU/mL in *Ae. aegypti* and from 5.8 to 6.0 log_10_ PFU/mL in *Ae. albopictus* (Fig 3D, S10 Table). Titers in leg samples ranged from 5.4 to 5.6 log_10_ PFU/mL in *Ae. aegypti* and from 5.1 to 5.5 log_10_ PFU/mL in *Ae. albopictus* (Fig 3E, S10 Table). Titers in saliva samples were overall lower, with a relatively narrow range in *Ae. aegypti* (3.6-4.0 log_10_ PFU/mL) and a broader range in *Ae. albopictus* (2.8-4.2 log_10_ PFU/mL) (Fig 3F, S10 Table).

## 4. Discussion

This study evaluated the vector competence of six mosquito species for MADV by assessing the presence of viral RNA and infectious particles in different biological samples from mosquitoes, including saliva. In addition, we assessed whether *Ae. taeniorhynchus* exhibited differential vector competence for two MADV strains and evaluated temporal infection dynamics in *Ae. aegypti* and *Ae. albopictus*.

Five of the six mosquito species tested had MADV RNA and infectious virus particles in their saliva following oral exposure indicating that MADV may be compatible with, and potentially transmitted by, a broad range of mosquito vectors in the field. As expected for arbovirus replication dynamics in mosquitoes, rates declined from body to legs to saliva, reflecting known biological barriers encountered by arboviruses within mosquito tissues [16,18,19]. The agreement between RT-qPCR and plaque assay results (where all but one RT-qPCR-positive sample yielded plaques, while none of the negative samples did) validated our molecular assay.

MADV-PAN body and leg infection probabilities varied across the six mosquito species at 14 dpe, likely reflecting species-specific midgut infection and escape barriers. Conversely, saliva infection probabilities were more uniform among competent species, although the limited number of positive saliva samples may have reduced power to detect differences.

The absence of positive saliva samples in *Cx. quinquefasciatus*, seen in both replicates, indicates that this species may not be competent for MADV-PAN. This species also had the lowest infection probabilities in body and legs, suggesting limited ability of the virus to infect and disseminate within its tissues.

Among the competent species, *Cx. tarsalis* is an ornithophilic mosquito that sporadically feeds on mammals. *Culex tarsalis* is a major West Nile virus vector and an important vector for western equine encephalitis in North America [20]. Although this species is not found in MADV-endemic areas, its potential to transmit MADV becomes relevant if the virus is introduced in regions within its range, particularly if birds are shown to participate in MADV transmission cycles. Although ground-dwelling mammals, such as rodents, are considered the putative primary reservoirs of MADV based on analysis of evolutionary patterns and field data [21,22], antibodies to MADV have been detected in some bird species, generally at low titers [7,11], and the virus has been isolated from a limited number of birds [11]. These observations indicate that birds can be exposed to and infected with MADV, although their role in transmission remains unclear.

*Culex coronator*, also competent for MADV, is widespread throughout the Americas [23–27]. This species has been implicated as a vector of certain arboviruses [28], but it is usually not included in disease surveillance testing. While *Cx. coronator* has generally been associated with sylvatic habitats, there are reports of its presence in urban and peri-urban areas [29,30]. Interestingly, *Cx. coronator* from Peru did not transmit MADV in the study by Turell *et al*. [14] – this discrepancy may be due to differences in mosquito and virus strains used in each study.

*Aedes aegypti*, another MADV-competent species in our study, is a major vector of arboviruses in urban and peri-urban environments due to its strong anthropophilic behavior [31,32]. This behavior makes its involvement in arbovirus enzootic transmission cycles unlikely. However, this mosquito is also present in rural areas, and host-feeding behavior can vary geographically, with some populations occasionally feeding on non-human mammals [32–34]. Given this variability and the species’ broad distribution throughout the Americas, its potential role as a bridge vector in MADV-endemic regions cannot be entirely ruled out.

MADV infection probabilities in body, legs, and saliva were assessed over time in *Ae. aegypti* and *Ae. albopictus*. *Aedes albopictus* was included in this study due to its global distribution [35,36], wide host range [32,35], and potential role as a bridge vector for arboviruses [37]. The temporal infection dynamics differed sharply between these species. In *Ae. aegypti*, infection probabilities of body, legs and saliva increased over time. In *Ae. albopictus*, infection probabilities increased from 3 to 7 dpe but declined thereafter across all sample types, with no saliva samples testing positive at 21 dpe. Regardless of the mechanisms, which warrants further investigations, the observed infection dynamics of MADV-PAN in these two species highlight the need to account for temporal variation in mosquito-virus interactions when modeling transmission, as they may help explain spatiotemporal heterogeneity in arboviral disease transmission patterns. For instance, if a temporal decrease in transmission rates shortens the period during which mosquitoes carry infectious virus in saliva in a given species, as observed here in *Ae. albopictus* infected with MADV, this could lead to reduced arbovirus transmission in areas where this species predominates. Overall, infection patterns of arboviruses in mosquito tissues vary in the literature and appear to depend on mosquito and virus species and strains, bloodmeal titers, and environmental factors [38–42].

*Aedes taeniorhynchus*, also called the black salt marsh mosquito, was competent for both MADV-PAN and MADV-BR. While body, leg, and saliva infection probabilities for MADV-PAN were similar to those of other species, probabilities showed a higher trend for MADV-BR. Although not statistically significant in leg and saliva samples, this trend was consistent across biological sample types and replicates, suggesting a true biological difference between MADV-BR and MADV-PAN. *Aedes taeniorhynchus* is widely distributed throughout coastal and some inland regions of the Americas and is capable of exploiting diverse habitats [43–49]. It has a broad vertebrate host range [50,51] and although typically considered a nuisance species, it is competent for several medically important arboviruses [43,44,52–61], including the North American EEEV [54], and the Venezuelan equine encephalitis virus [55], which co-circulates with MADV in some regions [4]. Although MADV was isolated from *Ae. taeniorhynchus* during an equine epizootic in Brazil in 1960 [62], the transmission potential of this species had not been experimentally confirmed. Our results, combined with ecological features of *Ae. taeniorhynchus* and the prior isolation of MADV from field-collected specimens [62], support the role of this species as a potential amplification and bridge vector during outbreaks.

Overall, the findings in *Ae. aegypti*, *Ae. albopictus*, and *Ae. taeniorhynchus* reinforce the need to investigate species- and strain-specific mosquito-virus interactions more thoroughly.

This study has limitations. For instance, while four species were tested using F0-F3 generations to better reflect field conditions, two species (*Cx. tarsalis* and *Cx. quinquefasciatus*) came from long-term established colonies, which may limit the generalizability of the results to wild populations. In addition, the artificial oral infection method used may have underestimated transmission rates, as numerous studies have shown that in vivo feeding leads to higher transmission rates than in vitro feeding [63,64]. Finally, we were unable to include *Culex* (*Melanoconion*) spp. – considered the primary candidates for enzootic MADV transmission (1, 12) – due to colonization difficulties and limited field collections. We encourage the inclusion of this group in future vector competence studies with MADV.

Despite these limitations, our study provides new insights into the transmission potential of mosquito species found in the United States, including those broadly distributed in the Americas (e.g., *Ae. taeniorhynchus*) and globally (e.g., *Ae. aegypti* and *Ae. albopictus*), for MADV. Although this pathogen remains poorly studied, it has been associated with high morbidity and mortality rates in equines and with severe disease and death in humans [1]. Identifying mosquito species capable of biologically transmitting MADV is essential for anticipating potential emergence events, particularly as the virus expands geographically. Studies like ours are critical for understanding arbovirus emergence and for risk assessment, more so when integrated with ecological and field data.

## Supporting information

S1 Table Infection probabilities of body, legs and saliva collected at 14 days-post exposure from different mosquito species exposed to Madariaga virus (strain Panama). Least-squares means of infection probabilities and 95% confidence intervals were estimated using logistic regression models.
(DOCX)

S2 Table Odds ratios derived from least squares means of body, legs, and saliva infection probabilities in mosquitoes infected with Madariaga virus (strain Panama). Samples were collected at 14 days-post exposure.
(DOCX)

S3 Table Pairwise comparisons of Madariaga virus (strain Panama) infection probability in saliva samples between *Culex quinquefasciatus* and other mosquito species. Samples were collected at 14 days-post exposure.
(DOCX)

S4 Table Infection probabilities of body, legs and saliva collected from *Aedes taeniorhynchus* exposed to Madariaga strain Panama (MADV-PAN) or Madariaga virus strain Brazil (MADV-BR), at 14 days-post exposure. Least-squares means of infection probabilities and 95% confidence intervals were estimated using logistic regression models.
(DOCX)

S5 Table Odds ratios derived from least squares means of the infection probabilities of Madariaga virus strain Panama (MADV-PAN) and Madariaga virus strain Brazil (MADV-BR), in body, legs and saliva collected from *Aedes taeniorhynchus* at 14 days-post exposure. (DOCX)

S6 Table Infection probabilities of body, legs and saliva collected from *Aedes aegypti and Aedes albopictus* infected with Madariaga (strain Panama), at 3, 7, 14, and 21 days-post exposure (dpe). Least-squares means of infection probabilities and 95% confidence intervals were estimated using logistic regression models.
(DOCX)

S7 Table Odds ratios derived from least squares means of body, legs, and saliva infection probabilities in *Aedes aegypti* and *Aedes albopictus* infected with Madariaga virus (strain Panama), at 3, 7, 14, and 21 days-post exposure (dpe). (DOCX)

S8 Table Mean log_10_-transformed plaque forming units per mL (PFU/mL) of Madariaga virus (strain Panama), in body, leg and saliva samples collected from six mosquito species at 14 days-post exposure. (DOCX)

S9 Table Mean log_10_-transformed plaque forming units per mL (PFU/mL) of Madariaga virus strain Panama (MADV-PAN) or Madariaga virus strain Brazil (MADV-BR), in body, leg and saliva samples collected from *Aedes taeniorhynchus* at 14 days-post exposure. (DOCX)

S10 Table Mean log_10_-transformed plaque forming units per mL (PFU/mL) of Madariaga virus strain Panama (MADV-PAN), in body, leg and saliva samples collected from *Aedes aegypti* and *Aedes albopictus* at 3, 7, 14, and 21 days-post exposure. (DOCX)
