# Supplementary material for: Transmission potential of Culex and Aedes species for Madariaga virus, a member of the eastern equine encephalitis virus complex
Source: PLoS Negl Trop Dis. 2026 May 12;20(5):e0013516. doi: 10.1371/journal.pntd.0013516 (PMC13189421; doi:10.1371/journal.pntd.0013516)
Supplement: S1 Table — Least-squares means of infection probabilities and 95% confidence intervals were estimated using logistic regression models. (DOCX) [file pntd.0013516.s001.docx]

**S1 Table.** Infection probabilities of body, legs and saliva collected at 14 days-post exposure from different mosquito species exposed to Madariaga virus (strain Panama). Least-squares means of infection probabilities and 95% confidence intervals were estimated using logistic regression models.

| **Mosquito species** | **Infection probability [95% CI]^1^** | | |
| --- | --- | --- | --- |
|  | **Body** | **Legs** | **Saliva** |
| *Aedes aegypti* | 0.622 [0.461-0.759] | 0.328 [0.2-0.489] | 0.201 [0.102-0.357] |
| *Aedes albopictus* | 0.297 [0.175-0.457] | 0.161 [0.077-0.306] | 0.1 [0.038-0.242] |
| *Aedes taeniorhynchus* | 0.412 [0.213-0.645] | 0.345 [0.157-0.598] | 0.104 [0.025-0.349] |
| *Culex coronator* | 0.23 [0.118-0.4] | 0.151 [0.068-0.301] | 0.133 [0.052-0.303] |
| *Culex tarsalis* | 0.334 [0.226-0.462] | 0.302 [0.199-0.431] | 0.116 [0.056-0.226] |
| *Culex quinquefasciatus*^2^ | 0.15 [0.08-0.265] | 0.033 [0.008-0.124] | - |
| ^1^Logistic regression models with a binomial distribution and logit link were used to estimate infection probabilities (body, legs, saliva). The fixed effect was ‘mosquito species’. Covariates included ‘bloodmeal titer’ and ‘replicate’; however, ‘replicate’ was removed from the final model as it did not significantly predict the outcome. Model outputs are presented as least-squares means of infection probabilities with 95% confidence intervals (CIs).  ^2^*Culex quinquefasciatus* had no positive saliva and was excluded from the saliva model. | | | |
