# Supplementary material for: Transmission potential of Culex and Aedes species for Madariaga virus, a member of the eastern equine encephalitis virus complex
Source: PLoS Negl Trop Dis. 2026 May 12;20(5):e0013516. doi: 10.1371/journal.pntd.0013516 (PMC13189421; doi:10.1371/journal.pntd.0013516)
Supplement: S2 Table — Samples were collected at 14 days-post exposure. (DOCX) [file pntd.0013516.s002.docx]

**S2 Table**. Odds ratios derived from least squares means of body, legs, and saliva infection probabilities in mosquitoes infected with Madariaga virus (strain Panama). Samples were collected at 14 days-post exposure.

| **Mosquito species** | **Reference species** | **Body** | | **Leg** | | **Saliva** | |
| --- | --- | --- | --- | --- | --- | --- | --- |
|  |  | **Odds ratio**  **[95% CI]** | **p-value** | **Odds ratio**  **[95% CI]** | **p-value** | **Odds ratio**  **[95% CI]** | **p-value** |
| *Aedes albopictus* | *Aedes taeniorhynchus* | 0.6 [0.18-2.06] | 0.4179 | 0.36 [0.09-1.47] | 0.1549 | 0.96 [0.14-6.62] | 0.9683 |
|  | *Culex coronator* | 1.41 [0.51-3.95] | 0.5072 | 1.08 [0.33-3.49] | 0.8999 | 0.73 [0.17-3.04] | 0.6603 |
|  | *Culex quinquefasciatus*^1^ | 2.39 [0.88-6.44] | 0.0855 | 5.56 [1.08-28.76] | *0.0407* | - | - |
|  | *Culex tarsalis* | 0.84 [0.35-2.02] | 0.6999 | 0.44 [0.16-1.21] | 0.1107 | 0.96 [0.14-6.62] | 0.9683 |
|  | *Aedes aegypti* | 0.26 [0.1-0.66] | *0.0046* | 0.39 [0.14-1.12] | 0.0800 | 0.73 [0.17-3.04] | 0.6603 |
| *Aedes taeniorhynchus* | *Culex coronator* | 2.34 [0.6-9.21] | 0.2212 | 2.97 [0.67-13.21] | 0.1527 | 0.76 [0.1-5.89] | 0.7877 |
|  | *Culex quinquefasciatus*^1^ | 3.96 [1.21-12.9] | *0.0227* | 15.31 [2.61-89.73] | *0.0026* | - | - |
|  | *Culex tarsalis* | 1.4 [0.47-4.13] | 0.5451 | 1.22 [0.37-3.95] | 0.7442 | 0.88 [0.16-4.79] | 0.8827 |
|  | *Aedes aegypti* | 0.43 [0.13-1.42] | 0.1645 | 1.08 [0.29-3.94] | 0.9106 | 0.46 [0.08-2.8] | 0.3994 |
| *Culex coronator* | *Culex quinquefasciatus*^1^ | 1.69 [0.57-4.95] | 0.3395 | 5.16 [0.98-27.28] | 0.0534 | - | - |
|  | *Culex tarsalis* | 0.6 [0.23-1.57] | 0.2949 | 0.41 [0.14-1.17] | 0.0946 | 1.17 [0.31-4.39] | 0.8188 |
|  | *Aedes aegypti* | 0.18 [0.07-0.5] | *0.0010* | 0.36 [0.12-1.06] | 0.0632 | 1.17 [0.31-4.39] | 0.8188 |
| *Culex quinquefasciatus*^1^ | *Culex tarsalis* | 0.35 [0.14-0.86] | *0.0225* | 0.08 [0.02-0.36] | *0.0012* | - | - |
|  | *Aedes aegypti* | 0.11 [0.04-0.28] | *<0.0001* | 0.07 [0.01-0.34] | *0.0010* | - | - |
| *Culex tarsalis* | *Aedes aegypti* | 0.31 [0.13-0.71] | *0.0063* | 0.89 [0.37-2.12] | 0.7842 | 0.52 [0.17-1.62] | 0.2618 |
| Logistic regression models were used to estimate infection probabilities in body, legs, and saliva. The fixed effect was ‘mosquito species’. Covariates included ‘bloodmeal titer’ and ‘replicate’; however, ‘replicate’ was removed from the final model as it did not significantly predict the outcome. Odds ratios (ORs) were derived from post hoc pairwise comparisons between groups. ORs >1 indicate higher odds, whereas ORs <1 indicate lower odds of infection probability relative to the reference species. Results are presented as ORs with 95% confidence intervals (CIs) and corresponding p-values.  ^1^For *Culex quinquefasciatus*, ORs for saliva were not estimated due to the absence of positive saliva samples in the molecular assay. | | | | | | | |
