# Supplementary material for: Transmission potential of Culex and Aedes species for Madariaga virus, a member of the eastern equine encephalitis virus complex
Source: PLoS Negl Trop Dis. 2026 May 12;20(5):e0013516. doi: 10.1371/journal.pntd.0013516 (PMC13189421; doi:10.1371/journal.pntd.0013516)
Supplement: S3 Table — Samples were collected at 14 days-post exposure. (DOCX) [file pntd.0013516.s003.docx]

**S3 Table.** Pairwise comparisons of Madariaga virus (strain Panama) infection probability in saliva samples between *Culex quinquefasciatus* and other mosquito species. Samples were collected at 14 days-post exposure.

| **Species 1** | **Species 2** |  | **Fisher’s exact test^1^** |  |
| --- | --- | --- | --- | --- |
|  |  | **Raw p-value** | **Bonferroni-adjusted p-value** | **FDR-adjusted p-value** |
| *Culex quinquefasciatus* | *Aedes aegypti* | *0.0004* | *0.0020* | *0.0020* |
|  | *Aedes albopictus* | *0.0233* | 0.1165 | *0.0291* |
|  | *Aedes taeniorhynchus* | *0.0299* | 0.1495 | *0.0299* |
|  | *Culex coronator* | *0.0074* | *0.0370* | *0.0185* |
|  | *Culex tarsalis* | *0.0130* | 0.0650 | *0.0217* |
| ^1^For *Culex quinquefasciatus* only, pairwise comparisons were performed using Fisher’s exact tests based on the original binary data (positive vs. negative saliva samples). Bonferroni and false discovery rate (FDR) corrections were applied to account for multiple comparisons. Adjusted p-values are shown. P-values in italics indicate statistical significance. | | | | |
