# Supplementary material for: Transmission potential of Culex and Aedes species for Madariaga virus, a member of the eastern equine encephalitis virus complex
Source: PLoS Negl Trop Dis. 2026 May 12;20(5):e0013516. doi: 10.1371/journal.pntd.0013516 (PMC13189421; doi:10.1371/journal.pntd.0013516)
Supplement: S4 Table — Least-squares means of infection probabilities and 95% confidence intervals were estimated using logistic regression models. (DOCX) [file pntd.0013516.s004.docx]

**S4 Table.** Infection probabilities of body, legs and saliva collected from *Aedes taeniorhynchus* exposed to Madariaga strain Panama (MADV-PAN) or Madariaga virus strain Brazil (MADV-BR), at 14 days-post exposure. Least-squares means of infection probabilities and 95% confidence intervals were estimated using logistic regression models.

| **MADV Strain** | **Infection probability [95% CI]^1^** | | |
| --- | --- | --- | --- |
|  | **Body** | **Legs** | **Saliva** |
| MADV-PAN | 0.374 [0.208-0.576] | 0.268 [0.13-0.474] | 0.110 [0.034-0.299] |
| MADV-BR | 0.776 [0.624-0.879] | 0.459 [0.31-0.615] | 0.298 [0.175-0.460] |
| ^1^Logistic regression models with a binomial distribution and logit link were used to estimate infection probabilities (body, legs, saliva). The fixed effect was ‘virus strain’. Covariates included ‘bloodmeal titer’ and ‘replicate’; however, ‘replicate’ was removed from the final model as it did not significantly predict the outcome. Model outputs are presented as least-squares means of infection probabilities with 95% confidence intervals (CIs). | | | |
