# Supplementary material for: Transmission potential of Culex and Aedes species for Madariaga virus, a member of the eastern equine encephalitis virus complex
Source: PLoS Negl Trop Dis. 2026 May 12;20(5):e0013516. doi: 10.1371/journal.pntd.0013516 (PMC13189421; doi:10.1371/journal.pntd.0013516)
Supplement: S5 Table — (DOCX) [file pntd.0013516.s005.docx]

**S5 Table.** Odds ratios derived from least squares means of the infection probabilities of Madariaga virus strain Panama (MADV-PAN) and Madariaga virus strain Brazil (MADV-BR), in body, legs and saliva collected from *Aedes taeniorhynchus* at 14 days-post exposure.

| **Virus strain** | **Reference strain** | **Body** | | **Leg** | | **Saliva** | |
| --- | --- | --- | --- | --- | --- | --- | --- |
|  |  | **Odds ratio [95% CI]** | **p-value** | **Odds ratio [95% CI]** | **p-value** | **Odds ratio [95% CI]** | **p-value** |
| MADV-BR | MADV-PAN | 5.8 [1.85-18.14] | *0.0030* | 2.32 [0.77-6.97] | 0.1326 | 3.45 [0.8-14.79] | 0.0946 |
| Logistic regression models were used to estimate infection probabilities in body, legs, and saliva. The fixed effect was ‘virus strain’. Covariates included ‘bloodmeal titer’ and ‘replicate’; however, ‘replicate’ was removed from the final model as it did not significantly predict the outcome. Odds ratios (ORs) were derived from post hoc pairwise comparisons between groups. ORs >1 indicate higher odds, whereas ORs <1 indicate lower odds of infection probability relative to the reference strain. Results are presented as ORs with 95% confidence intervals (CIs) and corresponding p-values. | | | | | | | |
