# Supplementary material for: Transmission potential of Culex and Aedes species for Madariaga virus, a member of the eastern equine encephalitis virus complex
Source: PLoS Negl Trop Dis. 2026 May 12;20(5):e0013516. doi: 10.1371/journal.pntd.0013516 (PMC13189421; doi:10.1371/journal.pntd.0013516)
Supplement: S6 Table — Least-squares means of infection probabilities and 95% confidence intervals were estimated using logistic regression models. (DOCX) [file pntd.0013516.s006.docx]

**S6 Table.** Infection probabilities of body, legs and saliva collected from *Aedes aegypti and Aedes albopictus* infected with Madariaga (strain Panama), at 3, 7, 14, and 21 days-post exposure (dpe). Least-squares means of infection probabilities and 95% confidence intervals were estimated using logistic regression models.

| **Mosquito species** | **Dpe** | **Infection probability [95% CI]^1^** | | |
| --- | --- | --- | --- | --- |
|  |  | **Body** | **Legs** | **Saliva** |
| *Aedes aegypti* | 3 | 0.325 [0.198-0.483] | 0.05 [0.012-0.179] | 0.0 [0.0-1.0] |
|  | 7 | 0.325 [0.198-0.483] | 0.124 [0.052-0.267] | 0.049 [0.012-0.178] |
|  | 14 | 0.625 [0.467-0.761] | 0.349 [0.218-0.508] | 0.198 [0.102-0.351] |
|  | 21 | 0.826 [0.676-0.915] | 0.5 [0.349-0.651] | 0.324 [0.197-0.483] |
| *Aedes albopictus* | 3 | 0.274 [0.158-0.432] | 0.274 [0.158-0.432] | 0.049 [0.012-0.178] |
|  | 7 | 0.65 [0.492-0.781] | 0.274 [0.158-0.432] | 0.149 [0.068-0.295] |
|  | 14 | 0.299 [0.178-0.458] | 0.174 [0.085-0.324] | 0.099 [0.037-0.237] |
|  | 21 | 0.1 [0.038-0.238] | 0.05 [0.012-0.179] | 0.0 [0.0-1.0] |
| ^1^Logistic regression models with a binomial distribution and logit link were used to estimate infection probabilities (body, legs, saliva). The fixed effects were ‘mosquito species’, ‘dpe’, and “mosquito species x dpe’ interaction. Covariates included ‘bloodmeal titer’ and ‘replicate’; however, ‘replicate’ was removed from the final model as it did not significantly predict the outcome. Model outputs are presented as least-squares means of infection probabilities with 95% confidence intervals (CIs). | | | | |
