# Supplementary material for: Transmission potential of Culex and Aedes species for Madariaga virus, a member of the eastern equine encephalitis virus complex
Source: PLoS Negl Trop Dis. 2026 May 12;20(5):e0013516. doi: 10.1371/journal.pntd.0013516 (PMC13189421; doi:10.1371/journal.pntd.0013516)
Supplement: S7 Table — (DOCX) [file pntd.0013516.s007.docx]

**S7 Table.** Odds ratios derived from least squares means of body, legs, and saliva infection probabilities in *Aedes aegypti* and *Aedes albopictus* infected with Madariaga virus (strain Panama), at 3, 7, 14, and 21 days-post exposure (dpe).

| **Mosquito species** | **Reference species** | **Dpe** | **Body** | | **Legs** | | **Saliva** | |
| --- | --- | --- | --- | --- | --- | --- | --- | --- |
|  |  |  | **Odds ratio**  **[95% CI]** | **p-value** | **Odds ratio**  **[95% CI]** | **p-value** | **Odds ratio**  **[95% CI]** | **p-value** |
| *Aedes albopictus* | *Aedes aegypti* | 3 | 0.79 [0.3-2.06] | 0.6258 | 7.23 [1.48-35.44] | *0.0149* | -^1^ | -^1^ |
|  |  | 7 | 3.87 [1.52-9.84] | *0.0046* | 2.66 [0.82-8.59] | 0.1013 | 3.36 [0.63-17.95] | 0.1550 |
|  |  | 14 | 0.26 [0.1-0.65] | *0.0045* | 0.39 [0.14-1.12] | 0.0805 | 0.44 [0.12-1.62] | 0.2183 |
|  |  | 21 | 0.02 [0.01-0.09] | *<0.0001* | 0.05 [0.01-0.25] | *0.0002* | -^1^ | -^1^ |
| Logistic regression models were used to estimate infection probabilities in body, legs, and saliva. The fixed effects were ‘mosquito species’, ‘dpe’, and ‘mosquito species x dpe’ interaction. Covariates included ‘bloodmeal titer’ and ‘replicate’; however, ‘replicate’ was removed from the final model as it did not significantly predict the outcome. Odds ratios (ORs) were derived from post hoc pairwise comparisons between groups. ORs >1 indicate higher odds, whereas ORs <1 indicate lower odds of infection probability relative to the reference species. Results are presented as ORs with 95% confidence intervals (CIs) and corresponding p-values.  ^1^*Aedes aegypti* at 3 dpe and *Ae. albopictus* at 21 dpe had no positive saliva samples, therefore the ORs were not reliably estimated.  P-values in italics represent statistical significance. | | | | | | | | |
