# Supplementary material for: Transmission potential of Culex and Aedes species for Madariaga virus, a member of the eastern equine encephalitis virus complex
Source: PLoS Negl Trop Dis. 2026 May 12;20(5):e0013516. doi: 10.1371/journal.pntd.0013516 (PMC13189421; doi:10.1371/journal.pntd.0013516)
Supplement: S8 Table — (DOCX) [file pntd.0013516.s008.docx]

**S8 Table**: Mean log_10_-transformed plaque forming units per mL (PFU/mL) of Madariaga virus (strain Panama), in body, leg and saliva samples collected from six mosquito species at 14 days-post exposure.

| **Mosquito species** | **Mean Log_10_ MADV-PAN PFU/mL [95% CI]^1^** | | |
| --- | --- | --- | --- |
|  | **Body** | **Legs** | **Saliva** |
| *Aedes aegypti* | 5.51 [5.39-5.64] | 5.46 [5.33-5.58] | 3.93 [3.77-4.10] |
| *Aedes albopictus* | 5.90 [5.69-6.10] | 5.13 [4.93-5.33] | 3.74 [3.54-3.94] |
| *Aedes taeniorhynchus* | 5.76 [5.55-5.97] | 5.49 [5.30-5.68] | 4.29 [3.76-4.83] |
| *Culex coronator* | 5.33 [5.18-5.48] | 5.23 [5.09-5.38] | 4.05 [3.82-4.27] |
| *Culex tarsalis* | 5.18 [5.06-5.31] | 5.39 [5.27-5.51] | 4.15 [4.01-4.29] |
| *Culex quinquefasciatus*^2^ | 5.22 [5.08-5.36] | 5.34 [5.08-5.59] | - |
| ^1^Log_10_-transformed viral titers (PFU/mL) were analyzed using generalized linear models. Least-squares means of viral titers with 95% confidence intervals (CIs) were estimated from the models and are presented in the table.  ^2^*Culex quinquefasciatus* did not have any positive saliva samples in the molecular assay. | | | |
