# Supplementary material for: Transmission potential of Culex and Aedes species for Madariaga virus, a member of the eastern equine encephalitis virus complex
Source: PLoS Negl Trop Dis. 2026 May 12;20(5):e0013516. doi: 10.1371/journal.pntd.0013516 (PMC13189421; doi:10.1371/journal.pntd.0013516)
Supplement: S9 Table — (DOCX) [file pntd.0013516.s009.docx]

**S9 Table.** Mean log_10_-transformed plaque forming units per mL (PFU/mL) of Madariaga virus strain Panama (MADV-PAN) or Madariaga virus strain Brazil (MADV-BR), in body, leg and saliva samples collected from *Aedes taeniorhynchus* at 14 days-post exposure.

| **Mosquito species** | **Virus lineage** | **Mean Log_10_ MADV PFU/mL [95% CI]^1^** | | |
| --- | --- | --- | --- | --- |
|  |  | **Body** | **Legs** | **Saliva** |
| *Aedes taeniorhynchus* | MADV-PAN | 5.75 [5.62-5.88] | 5.49 [5.35-5.63] | 4.08 [3.75-4.42] |
|  | MADV-BR | 5.34 [5.26-5.42] | 5.16 [5.07-5.26] | 3.78 [3.62-3.94] |
| ^1^Log_10_-transformed viral titers (PFU/mL) were analyzed using generalized linear models. Least-squares means of viral titers with 95% confidence intervals (CIs) were estimated from the models and are presented in the table. | | | | |
