# Supplementary material for: Transmission potential of Culex and Aedes species for Madariaga virus, a member of the eastern equine encephalitis virus complex
Source: PLoS Negl Trop Dis. 2026 May 12;20(5):e0013516. doi: 10.1371/journal.pntd.0013516 (PMC13189421; doi:10.1371/journal.pntd.0013516)
Supplement: S10 Table — (DOCX) [file pntd.0013516.s010.docx]

**S10 Table**: Mean log_10_-transformed plaque forming units per mL (PFU/mL) of Madariaga virus strain Panama (MADV-PAN), in body, leg and saliva samples collected from *Aedes aegypti* and *Aedes albopictus* at 3, 7, 14, and 21 days-post exposure.

| **Mosquito species** | **Dpi** | **Mean Log_10_ MADV-PAN PFU/mL [95% CI]^1^** | | |
| --- | --- | --- | --- | --- |
|  |  | **Body** | **Legs** | **Saliva** |
| *Aedes aegypti* | 3 | 5.59 [5.42-5.77] | 5.36 [5.02-5.70] | -^2^ |
|  | 7 | 5.58 [5.40-5.76] | 5.57 [5.37-5.76] | 3.64 [2.75-4.54] |
|  | 14 | 5.51 [5.38-5.64] | 5.47 [5.36-5.59] | 3.97 [3.52-4.41] |
|  | 21 | 5.61 [5.50-5.72] | 5.44 [5.34-5.54] | 3.76 [3.41-4.11] |
| *Aedes albopictus* | 3 | 5.80 [5.56-6.03] | 5.32 [5.13-5.51] | 4.15 [3.22-5.09] |
|  | 7 | 5.78 [5.62-5.94] | 5.22 [5.06-5.37] | 2.80 [2.22-3.37] |
|  | 14 | 5.93 [5.70-6.17] | 5.13 [4.93-5.32] | 3.82 [3.18-4.45] |
|  | 21 | 5.95 [5.62-6.28] | 5.46 [5.22-5.70] | -^2^ |
| ^1^Log_10_-transformed viral titers (PFU/mL) were analyzed using generalized linear models. Least-squares means of viral titers with 95% confidence intervals (CIs) were estimated from the models.  ^2^*Aedes aegypti* at 3 dpi and *Ae. albopictus* at 21 dpi had no positive saliva samples. | | | | |
